# Host genetics and larval host plant modulate microbiome structure and evolution underlying the intimate insect–microbe–plant interactions in *Parnassius* species on the Qinghai‐Tibet Plateau

**DOI:** 10.1002/ece3.11218

**Published:** 2024-04-10

**Authors:** Chengyong Su, Tingting Xie, Lijun Jiang, Yunliang Wang, Ying Wang, Ruie Nie, Youjie Zhao, Bo He, Junye Ma, Qun Yang, Jiasheng Hao

**Affiliations:** ^1^ College of Life Sciences Anhui Normal University Wuhu China; ^2^ College of Physical Education Anhui Normal University Wuhu China; ^3^ Key Laboratory of Palaeobiology and Petroleum Stratigraphy, Center for Excellence in Life and Palaeoenvironment, Nanjing Institute of Geology and Paleontology Chinese Academy of Sciences Nanjing China; ^4^ Nanjing College University of Chinese Academy of Sciences Nanjing China

**Keywords:** gut microbiome, host genetics, host plant, *Parnassius*, Qinghai‐Tibet Plateau

## Abstract

Insects harbor a remarkable diversity of gut microbiomes critical for host survival, health, and fitness, but the mechanism of this structured symbiotic community remains poorly known, especially for the insect group consisting of many closely related species that inhabit the Qinghai‐Tibet Plateau. Here, we firstly analyzed population‐level 16S rRNA microbial dataset, comprising 11 *Parnassius* species covering 5 subgenera, from 14 populations mostly sampled in mountainous regions across northwestern‐to‐southeastern China, and meanwhile clarified the relative importance of multiple factors on gut microbial community structure and evolution. Our findings indicated that both host genetics and larval host plant modulated gut microbial diversity and community structure. Moreover, the effect analysis of host genetics and larval diet on gut microbiomes showed that host genetics played a critical role in governing the gut microbial beta diversity and the symbiotic community structure, while larval host plant remarkably influenced the functional evolution of gut microbiomes. These findings of the intimate insect–microbe–plant interactions jointly provide some new insights into the correlation among the host genetic background, larval host plant, the structure and evolution of gut microbiome, as well as the mechanisms of high‐altitude adaptation in closely related species of this alpine butterfly group.

## Graphical abstract

We firstly analyzed population‐level 16S rRNA microbial dataset, comprising 11 *Parnassius* species covering five subgenera, from 14 populations mostly sampled in mountainous regions across northwestern‐to‐southeastern China, and meanwhile clarified the relative importance of multiple factors on gut microbial community structure and evolution. Combined with the analyses of biomarkers, co‐occurrence pattern, and potential functions of gut microbes in butterfly species herein, our results jointly support the hypothesis of the coevolution between *Parnassius* species and gut microbes over long evolutionary timescales, providing a clue for the underlying insect–microbe–plant interactions and high‐altitude adaptation mechanisms. Our findings can help to resolve the major modulators of gut microbiome diversity and evolution in butterfly species, which have not been well studied in wild insects with varying degrees of ecological niche divergence and evolutionary relatedness, especially those inhabiting the QTP.

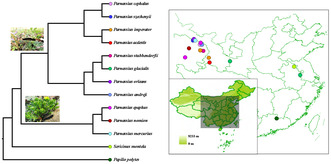

## 1 INTRODUCTION

Trillions of microbes can colonize the insect host's gut, forming a complex microecosystem and acting as an internalized “microbial organ” (Liu et al., 2021). Gut microbes are closely related to the insect host's physiology, morphology, behavior, and environmental adaptations, not only providing certain nutrients such as amino acids and vitamins required for growth and reproduction but also enhancing the host's resistance to external parasites, pathogens, and other unfavorable factors (Brown et al., 2020; Gould et al., 2018; Hosokawa & Fukatsu, 2020; Liu et al., 2021). In return, gut microbes can depend on their host to obtain a stable ecosystem and nutrients. Previous research suggested that host diet had a strong impact on the gut microbes, leading to similarities in gut microbiome among host species with different evolutionary backgrounds (Mallott & Amato, 2021; Nasvall et al., 2021). However, increasing studies have revealed that the impact of the host's diet on the gut microbes does not override the influence of the host's genetics (Donohue et al., 2022; Hammer et al., 2020; Huang et al., 2021; Mallott & Amato, 2021; Sanders et al., 2014; Weinstein et al., 2021). Consequently, the observed patterns of phylosymbiosis (Brooks et al., 2016), namely the microbial composition that can be predicted by host genetics, probably can establish causal links between the microbiome and the host's evolutionary trajectory (Mallott & Amato, 2021). To date, despite evidence that phylosymbiosis is mostly found under artificial conditions (Brooks et al., 2016; Hannula et al., 2019), the mechanism contributing to this pattern remains poorly understood, especially for the insect group, such as a genus consisting of many closely related species in natural environments, the alpine *Parnassius* butterflies on Qinghai‐Tibet Plateau (QTP), which exhibit phylogenetic discordance due to rapidly adaptive radiation.

Organisms on the QTP have often adaptively evolved to cope with extreme environmental conditions such as low temperatures, low oxygen, and high UV radiation (Han et al., 2021; Liu et al., 2021; Zhao & Li, 2017). In recent years, with the development of high‐throughput sequencing technology, gut microbiomics of animals has been widely used to explore the adaptive evolution of organisms on the QTP. Previous studies showed that the multiple factors influencing the composition and function of gut microbiota were complicated, and the specificity of gut microbiota might be closely related to the host's adaptation to specific habitats (Bapatla et al., 2018; Phalnikar et al., 2018; Wu et al., 2020). However, due to the complexity of microbial communities and difficulty in host captivity under natural conditions, the effects of gut microbes on the insect host's high‐altitude adaptation remain unexplained (Liu et al., 2021). As for the butterflies, only one or limited number of species under natural or artificial conditions have been reported (Minard et al., 2019; Nasvall et al., 2021; Ravenscraft et al., 2019).

The genus *Parnassius* is a typical alpine butterfly group, mainly distributed across the Holarctic, with its highest diversity on the Qinghai‐Tibet Plateau (QTP) and adjacent mountainous regions (including Xinjiang and Gansu, China). The majority of species in this genus often occur in sympatry and the same species could colonize at different localities, across a broad elevational range 2800–5200 m above sea level (Chou, 1994; Su et al., 2023; Wu, 2001). However, a few species also can secondarily colonize at relatively low‐altitude habitats, such as *Parnassius glacialis*, mainly distributed at about 200–1800 m above sea level in central‐to‐southeastern China. Species of this genus at larval stage feed mostly on the Crassulaceae and Saxifragaceae plants (core Saxifragales, including *Rhodiola*, *Sedum*, *Saxifraga*, etc.) for the subgenus *Parnassius*, or on the Papaveraceae (*Corydalis*) for the other subgenera of *Parnassius* (Allio et al., 2021). Previous studies have suggested that the genus *Parnassius* probably originated and experienced rapid radiation following the host plant shifts at about 17–13 million years ago during the middle‐to‐late Miocene, with the spatiotemporally evolutionary pattern correlating with the larval host plant's diversification, paleo‐geological, and paleo‐environmental changes on the QTP, supporting the underlying coevolutionary scenario between *Parnassius* and host plants (Allio et al., 2021; Condamine et al., 2018; Su et al., 2020; Tao et al., 2020; Zhao et al., 2022), and making them an ideal model for detecting the mechanism of the potential insect–microbe–plant interactions. Recently, extensive phylogenetic discordance (e.g., different tree topologies derived from different nuclear, or mitochondrial concatenated datasets) due to incomplete lineage sorting following the rapid radiation of this alpine butterfly group has been reported (He et al., 2023). However, in the process of spatiotemporal diversification, how and when the microbial community structure of this group is shaped, that is to say, it is shaped by host genetic background (phylosymbiosis) or by other factors is not yet addressed.

In the present study, the 16S rDNA V3–V4 region of the gut microbiota of 11 close‐related *Parnassius* species covering five of the six subgenera from 14 populations at 10 sites which occurred in sympatry and allopatry, and the other two papilionid species in China were firstly sequenced using high‐throughput sequencing technology. Based on the new population‐level genome re‐sequencing data and field investigation from our laboratory, we attempted to: (i) characterize and compare the gut microbiota among field‐collected adult butterfly species that occurred in sympatry and allopatry, (ii) determine factors (e.g., host genetics, geography, altitude, or larval host plant use) that could drive the variation in gut microbial communities for *Parnassius* species, and (iii) decipher biomarkers, co‐occurrence pattern, and potential functions of main gut microbiota, aiming to better understand the intimate insect–microbe–plant interactions under extreme environmental conditions on the QTP.

## 2 RESULTS

### 2.1 Sequencing data and general statistics of the gut microbiome composition

**FIGURE 1:**
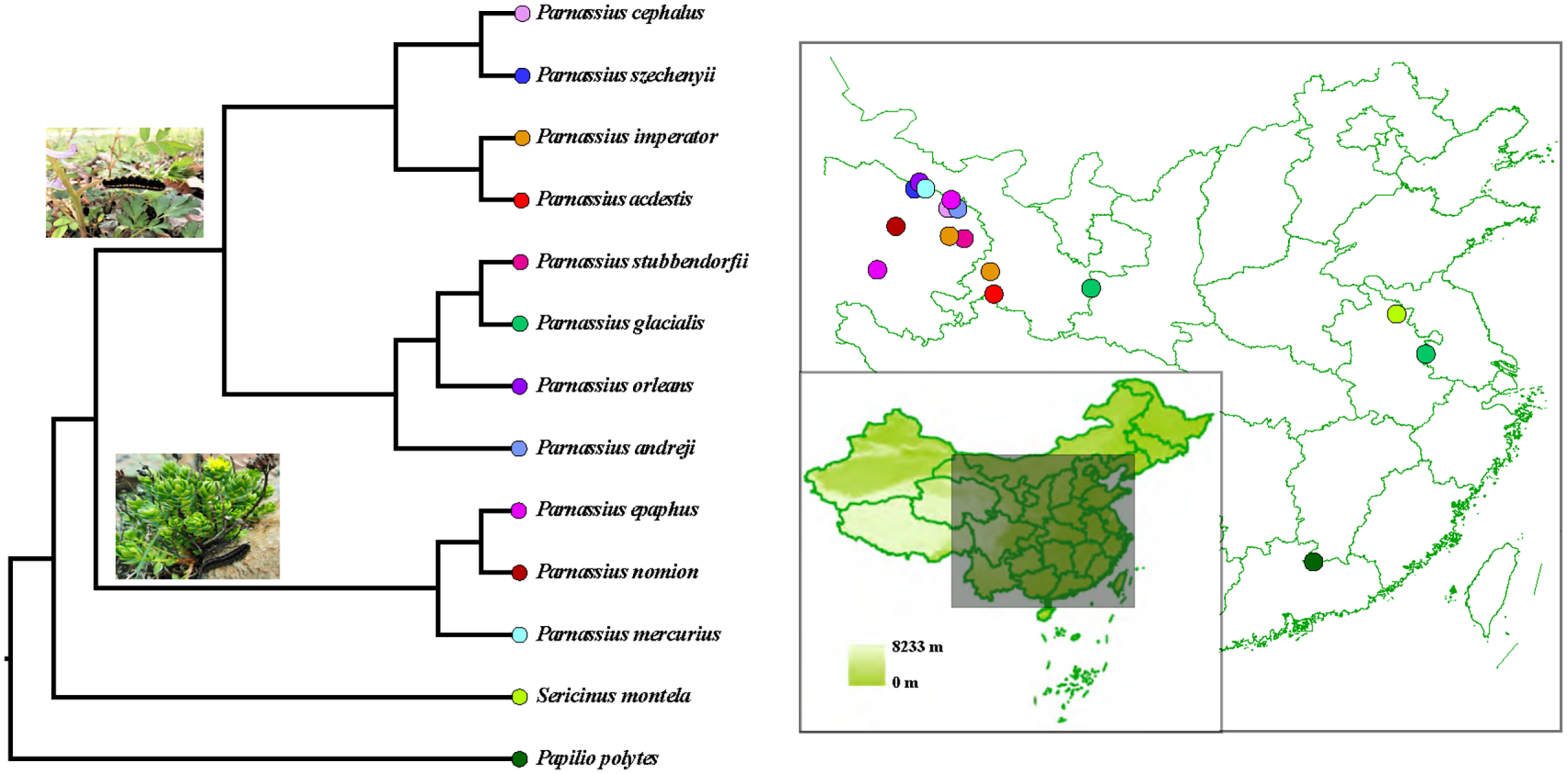
Phylogeny, geographic distribution, and larval diet of *Parnassius* species sampled in this study. The phylogeny was adapted from Allio et al. (2021). Different larval host plants for the subgenus *Parnassius* (*Rhodiola* in Crassulaceae, lower) and other subgenera in *Parnassius* (*Corydalis* in Papaveraceae, upper) were shown, respectively. Color for each solid circle on the terminal branch (left) represents the sampling site for each population, corresponding to that in the map (right).

A total of 75 samples (225 imago individuals) for 13 papilionid species from 16 populations at 12 sites were sampled (Table S1, Figure 1) and 7,220,338 raw reads were produced. After quality control, these samples were covered by a total of 5,864,963 reads. The mean sequencing depth per sample was 78,200 (ranging from 37,603 to 99,499) (Table S2). The mean percentage of valid sequences for all samples was 81.23% (ranging from 43.13% to 93.81%) (Table S2). Rarefaction curves suggested that the sequencing depth was sufficient to represent the microbial diversity (Figure S1). Good's coverage ranged from 97.2% to 100%, indicating that vast majority of microbial phylotypes were identified in all samples (Table S3). After rarefaction, 37,321 nonsingleton amplicon sequence variants (ASVs) for each sample were retained. More than 44 bacteria phyla and 1265 genera were detected in the gut microbiome of the 13 papilionid butterfly species after the chloroplastic, mitochondrial, and nonbacterial‐related ASVs were filtered out (Table S4).

**FIGURE 2:**
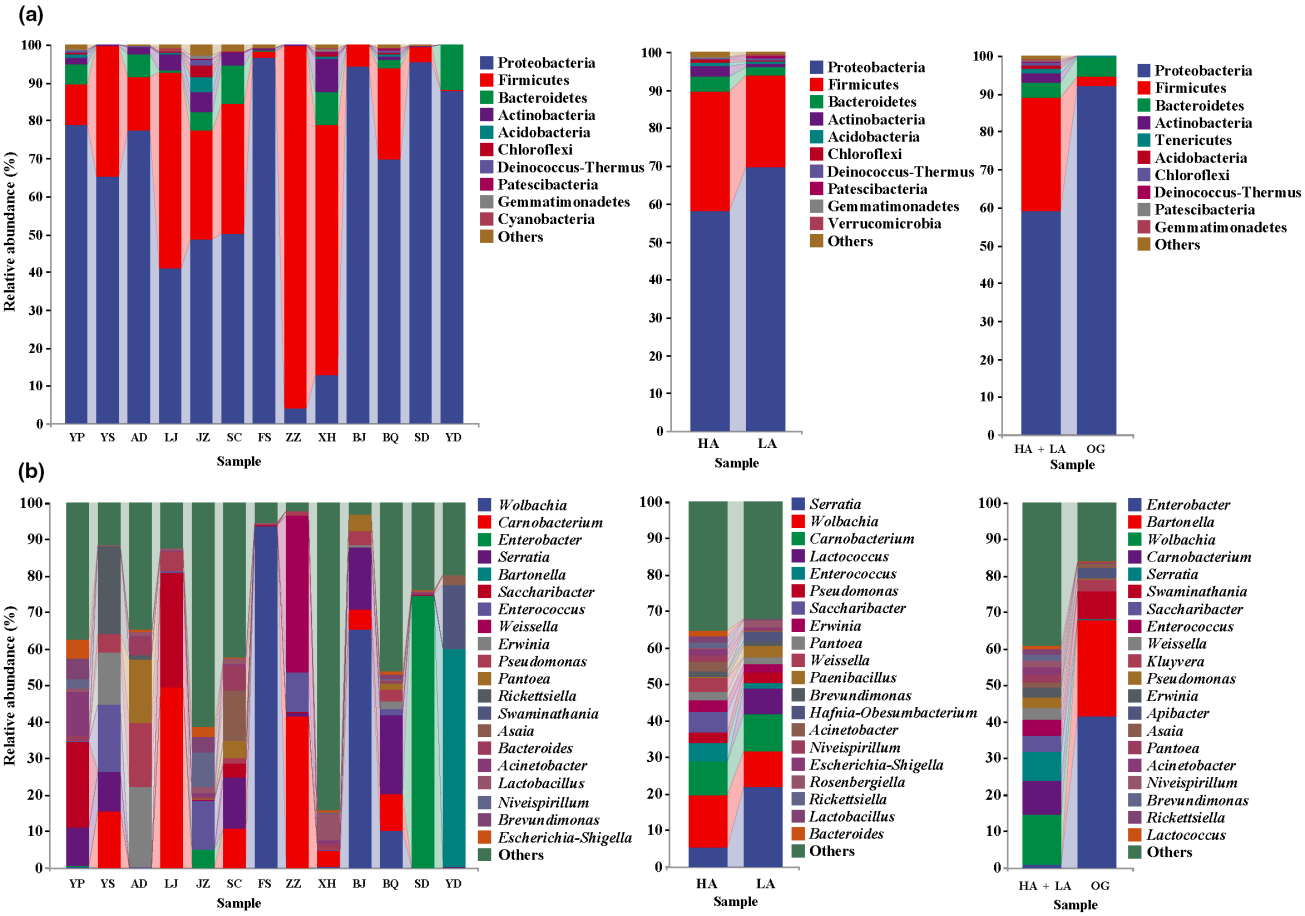
Relative abundance of the gut bacterial groups at the phylum (a) and genus (b) level. Phyla with the top 10 relative abundance and genera with the top 20 relative abundance were shown.

Generally, the relative abundance for each dominant microbial phylum and genus was largely species and population specific (Figure 2). However, the dominant microbial phyla were Proteobacteria, Firmicutes, Bacteroidetes, and Actinobacteria in both *Parnassius* and other two representative papilionid species (Figure 2a). The dominant microbial genera with the top 20 relative abundance are largely shared among butterfly species herein (e.g., *Enterococcus*, *Pseudomonas*, *Acinetobacter*, *Lactobacillus*, *Brevundimonas*, and *Escherichia*), and *Wolbachia*, *Carnobacterium*, *Saccharibacter*, *Erwinia*, *Rickettsiella*, and *Niveispirillum* were specifically identified in *Parnassius* species (Figure 2b).

### 2.2 Differences in microbial alpha diversity among species or populations

**FIGURE 3:**
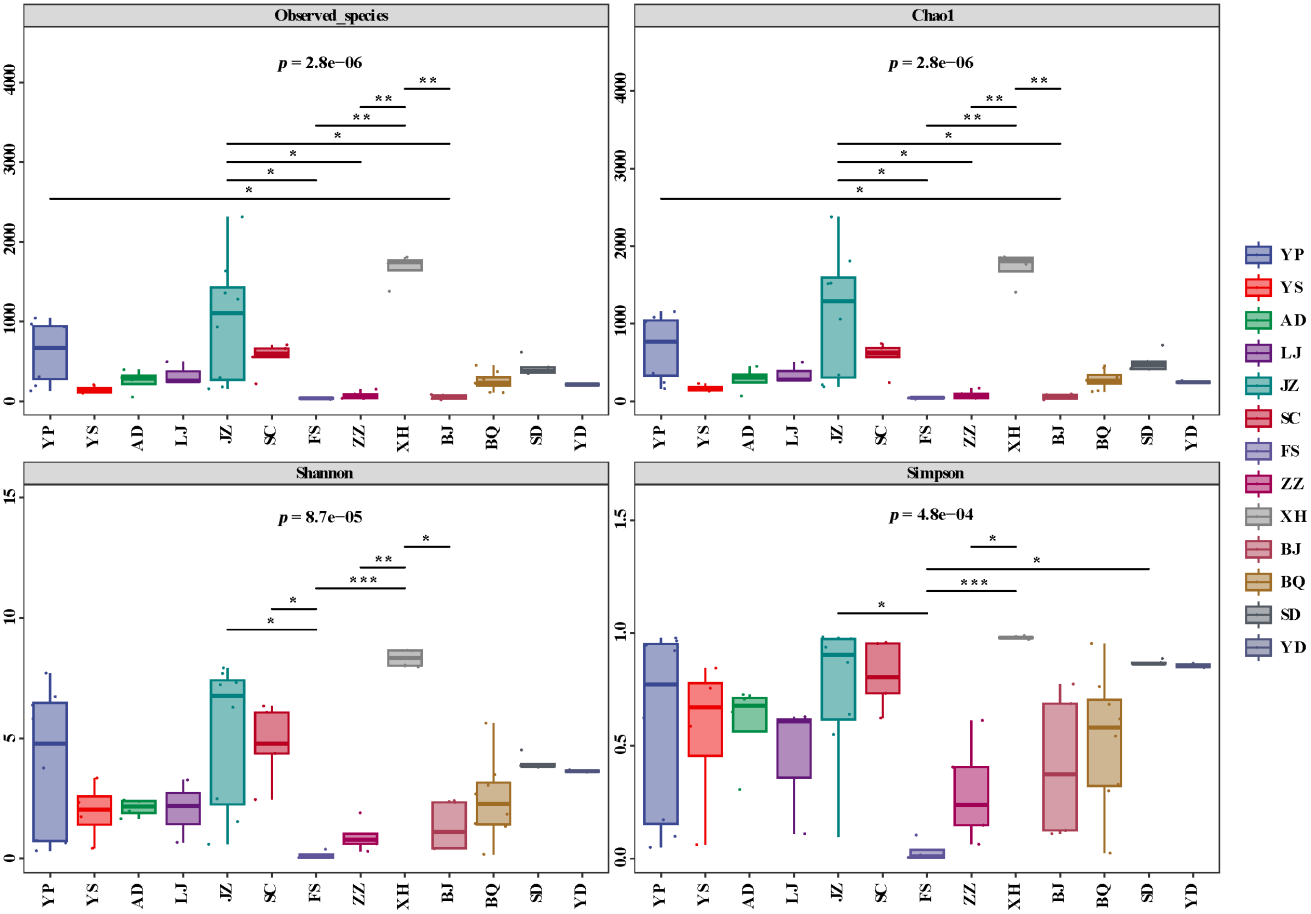
Comparisons of the microbial alpha diversity among host species.

Four indices (including observed species, Chao1, Shannon, and Simpson) were used to estimate the microbial alpha diversity (Table S2). Generally, significant differences in microbial alpha diversity were observed among multiple species (Kruskal–Wallis test, *p* < .001, Figure 3). In pairwise comparison, significantly higher alpha diversities for *P. imperator* (JZ) and *P. nomion* (XH) were detected when compared to those of *P. mercurius* (FS), *P. orleans* (ZZ), and *P. stubbendorfii* (BJ) (Figure 3, pairwise post hoc Dunn's test, *p* < .05).

**FIGURE 4:**
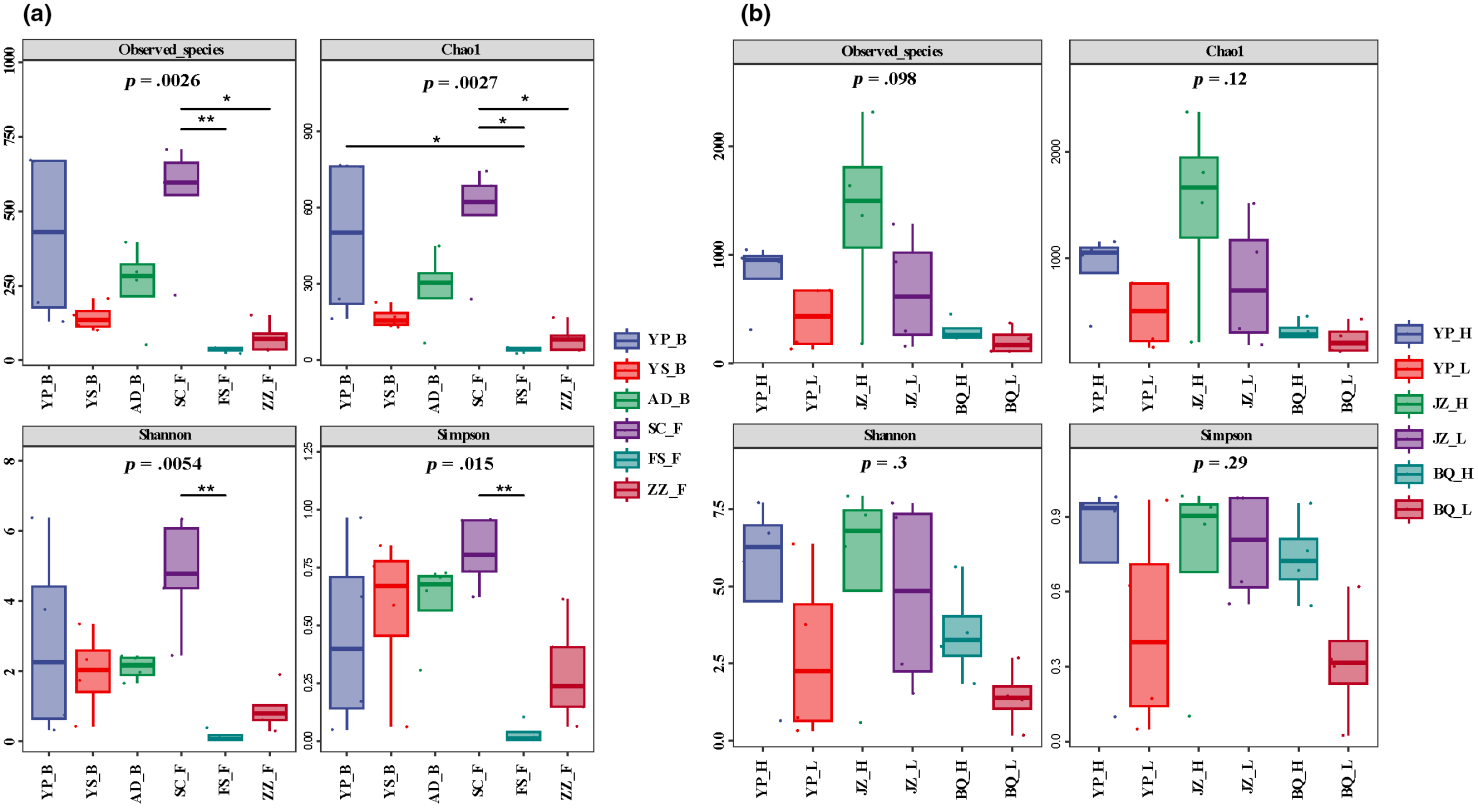
Comparisons of the microbial alpha diversity (a) for interspecific populations distributed in sympatry and (b) for intraspecific populations sampled in allopatry.

For different species distributed in sympatry, a significantly higher alpha diversity was detected for *P. szechenyii* (SC_F) samples (pairwise post hoc Dunn's test, *p* < .05), while no significant differences were found in other pairwise comparisons (Figure 4a). Results above indicated that interspecific variations in microbial alpha diversity were mostly species or population specific, regardless of the distributions of sympatry and allopatry. In addition, for intraspecific populations herein, no significant alpha diversity variation was observed (*p* > .05, Figure 4b).

### 2.3 Differences in microbial communities (beta diversity) among species in sympatry

**FIGURE 5:**
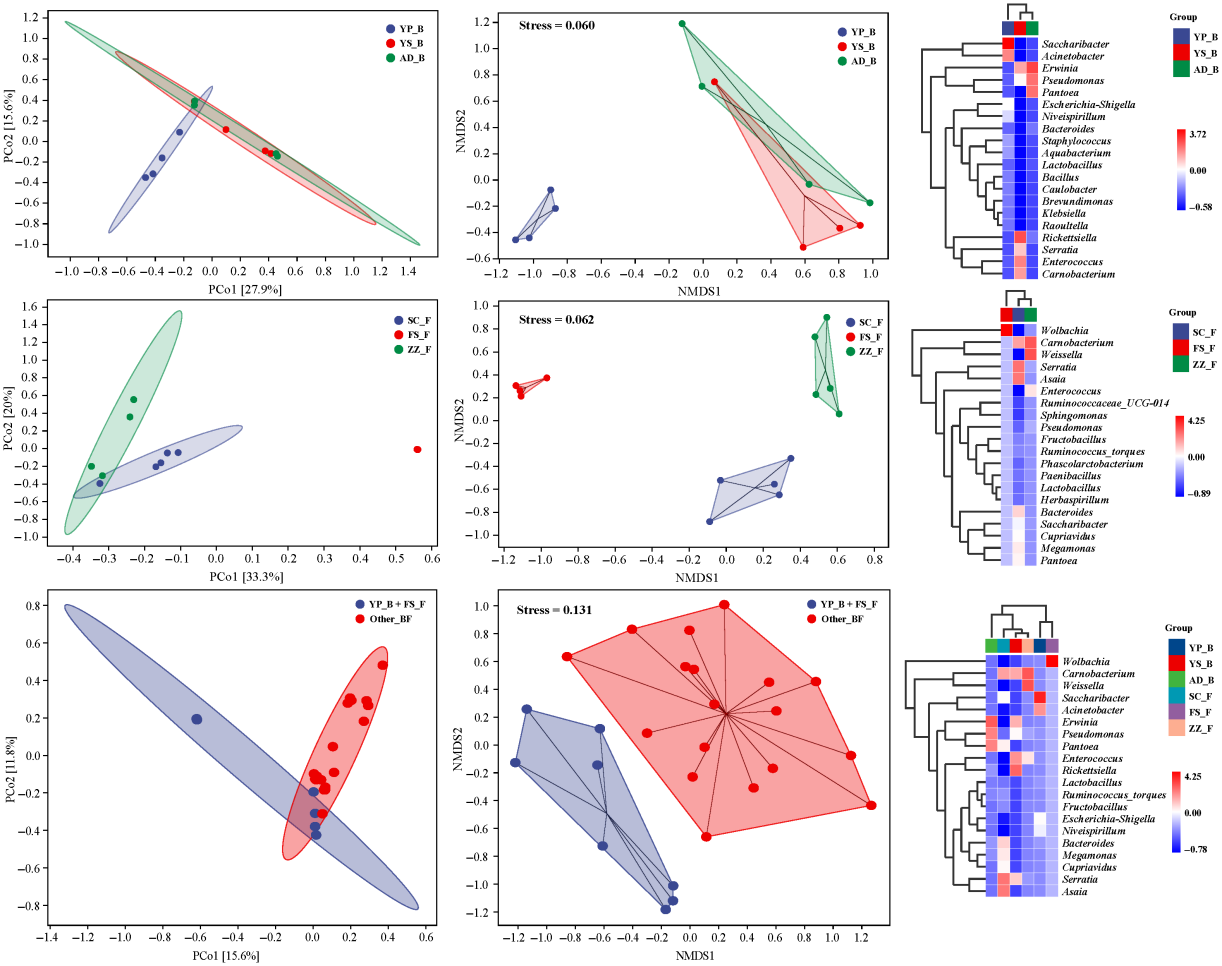
Comparisons of beta diversity among *Parnassius* species occurred in sympatry based on principal coordinate analysis (PCoA, left) and nonmetric multidimensional scaling analysis (NMDS, intermediate) using Bray–Curtis distance. Heatmaps (right) were made to compare the mean relative abundance of the top 20 genera among groups. Clustering pattern was based on the UPGMA hierarchical clustering algorithm using Euclidean distance of microbiome composition data as the distance metrics.

For three representative species sampled in Menyuan, Qinghai Province (Table S1), results based on the principal coordinate analysis (PCoA) and nonmetric multidimensional scaling analysis (NMDS) accompanied by the permutational multivariate analysis of variance (PERMANOVA) using four distance metrics generally showed significant variations in microbial communities between YP_B (in subgenus *Parnassius*, Crassulaceae as the main larval host plant) and other two species (YS_B and AD_B in other subgenera, Papaveraceae as the larval host plant) (Bray–Curtis, Jaccard, and weighted UniFrac: *p*_adj_ < .05, Figure 5). However, no significant variation was found between YS_B and AD_B in PERMANOVA for most of distance metrics (Bray–Curtis, weighted and unweighted UniFrac: *p*_adj_ > .05, Figure 5). A similar pattern was also found for another three sympatric species sampled at Qilian, Qinghai Province. Even if interspecific differences in microbial communities were detected, differences between species using the same plant as the larval food were relatively closer than those using different plants as the larval food.

Moreover, remarkably significant difference in microbial community structure between species in subgenus *Parnassius* (YP_B + FS_F) and those in other subgenera (Other_BF) was also detected in PERMANOVA based on most of distance metrics (Bray–Curtis, Jaccard, and weighted UniFrac: *p*_adj_ < .02, Figure 5). Heatmaps based on the mean relative abundance of the top 20 microbial genera revealed that YP_B still clustered with FS_F from the same subgenus (both using Crassulaceae as main host plant), rather than with other species (all using Papaveraceae as the host plant) sampled in the same locality. These results suggested that closely related allopatric species tend to harbor more similar gut microbes than distantly related sympatric species and that both host genetics and larval host plant could act as major drivers for the differences in microbial community structure. Moreover, only 8 of the top 20 microbial genera were found to be shared among species of two groups sampled in different localities above (Figure 5).

### 2.4 Differences in microbial communities for species in allopatry

**FIGURE 6:**
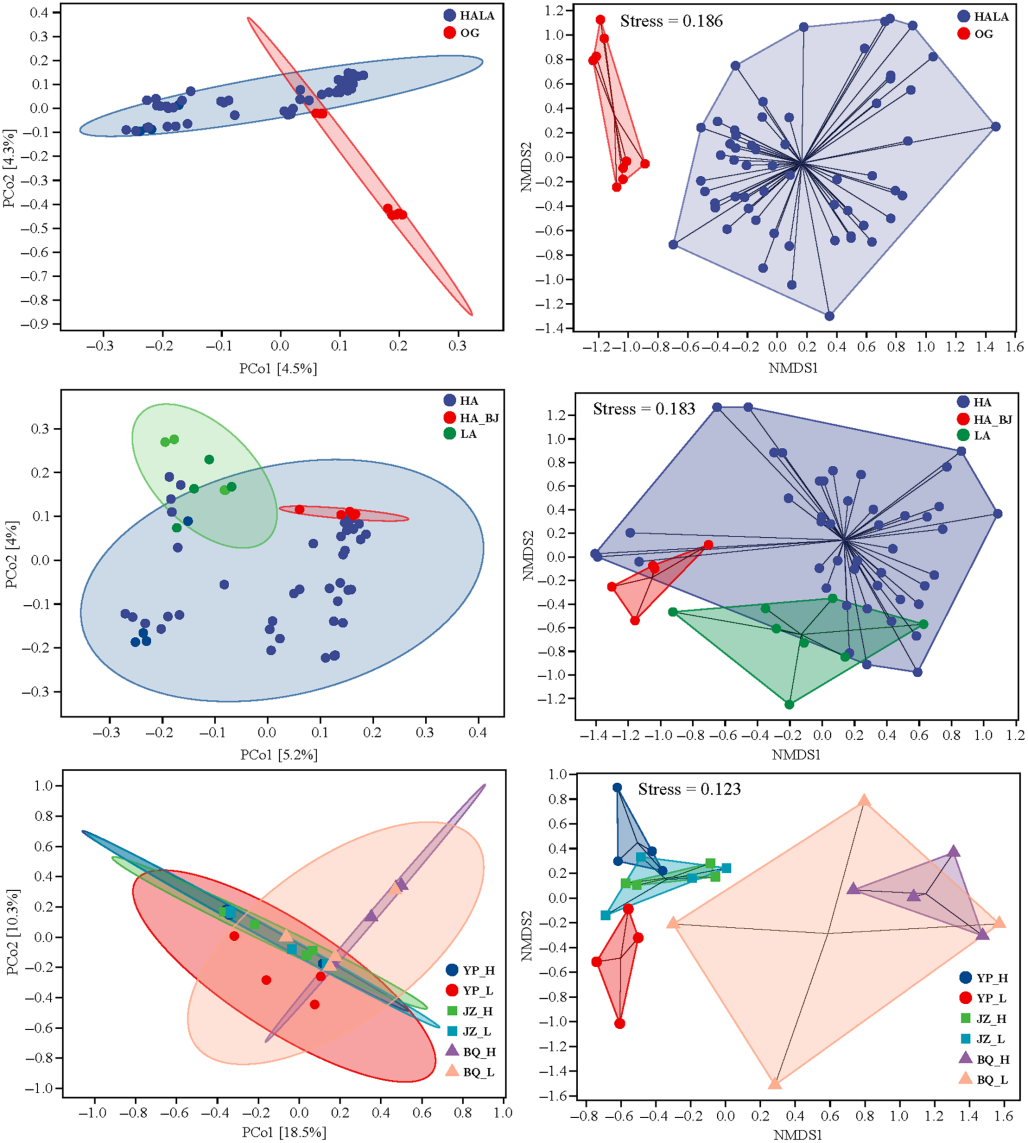
Comparisons of beta diversity among groups (upper and intermediate) or between intraspecific populations (lower) occurred in allopatry, based on PCoA (left) and NMDS (right) using Bray–Curtis distance.

As we predicted, significant difference in microbial community structure between *Parnassius* (including the group of HA and LA) and other papilionid species (the OG group) in allopatry was found based on the results of PCoA, NMDS, and PERMANOVA for all distance metrics (*p*_adj_ < .05, Figure 6). Similar pattern was also shown for different *Parnassius* species, especially between HA and LA groups in allopatry (Figure 6). However, for intraspecific populations in allopatry occurred in high‐, medium‐, and low‐altitude regions herein (e.g., YP_H vs. YP_L, JZ_H vs. JZ_L, and BQ_H vs. BQ_L, with geographical distances up to 336, 196, and 1152 km, respectively), no significant variations in microbial community structure were observed (PERMANOVA, all distance metrics: *p*_adj_ > .1), even for those sampled in different years (Table S1), probably indicating that the geographical distance had little effects on the beta diversity for intraspecific populations.

Moreover, although sampled at different sites with varied altitudes, YP_H and YP_B were still clustered together and differed significantly from other samples (YS_B and AD_B) collected at the same location (PERMANOVA, Bray–Curtis, and weighted and unweighted UniFrac: *p*_adj_ < .05, Figure S2). Similarly, although BQ_H and BQ_L were sampled in different years and locations at different altitudes, they were also clustered together (PERMANOVA, Bray–Curtis, and weighted and unweighted UniFrac: *p*_adj_ > .05), and were generally separated from other samples (ZZ_F and BJ) in the same subgenus (PERMANOVA, Bray–Curtis, Jaccard, and unweighted UniFrac: *p*_adj_ < .05, Figure S2). These results suggested that beta diversity of different intraspecific populations was generally lower than that among species, and the effects of species taxonomy associated with host genetics that resulted from the long‐term evolutionary history were likely to be stronger than those of elevation and geographic distribution, although the latter two also likely influenced microbial structure of other animal groups as shown in previous studies (Weinstein et al., 2021).

### 2.5 Factors contributing to the variation of gut microbial diversity and community structure for *Parnassius* species

**FIGURE 7:**
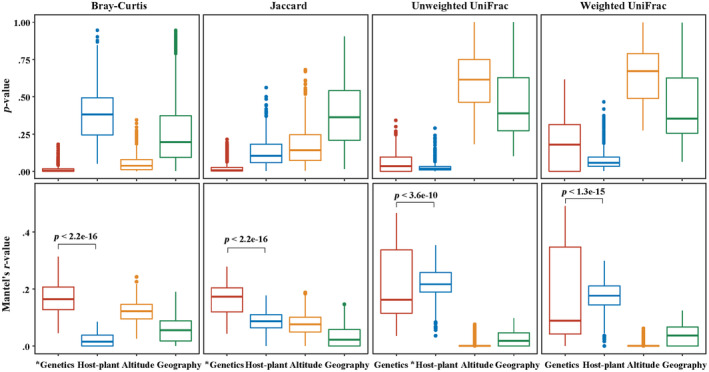
Results for partial Mantel tests between beta diversity (Bray–Curtis, Jaccard, unweighted UniFrac, or weighted UniFrac metrics) and four potential factors for wild *Parnassius* species, regardless of host phylogenetic distance. The boxplots show the distribution of values obtained when running partial Mantel tests on each of the 100 random dataset subsets. Asterisk denotes significance (*p* < .05 for most of dataset subsets). Box centerlines, edges, whiskers, and points signify the median, interquartile range (IQR), 1.5× IQR, and >1.5× IQR, respectively. Significant differences (Wilcoxon rank‐sum test) in partial Mantel correlations were shown between host genetics and larval diet dissimilarity.

To explore the independent effects of multiple factors on microbial community, partial Mantel tests were conducted in this study. For *Parnassius* species herein, different host species generally harbored distinct microbial communities (PERMANOVA, all distance metrics: *p*_adj_ < .05 for the average >75% of all pairwise comparisons) (Table S5). Partial Mantel tests also showed that more closely related host species generally harbored more similar microbial community structure (Figure 7, Table S6; controlling for geographic distance, altitude, or host plant dissimilarity, most distance metrics except the UniFrac metrics for the nuclear‐based single‐nucleotide variants [SNVs] dataset: *p* < .05), generally supporting the phylosymbiosis hypothesis for correlation‐based analysis above. Interestingly, larval host plant dissimilarity was more closely correlated with the variations of microbial beta diversity based on phylogenetically weighted metrics (unweighted and weighted UniFrac) than those based on taxonomy‐based metrics (Jaccard and Bray–Curtis, partial Mantel tests controlling for host genetic distance, geographic, or altitude dissimilarity). Significant differences in partial Mantel correlations were found between host genetics and larval diet dissimilarity (*p* < 3.6e‐10, Figure 7). All these suggested there existed a significant phylogenetic correlation between host plant and gut microbes, and that older microbial clades in deeper evolutionary history probably have stronger associations with host plant than younger clades, as demonstrated in some mammalian taxa (Donohue et al., 2022). Increasing either geographic distance or altitude dissimilarity mostly had no significant effect on microbial dissimilarity for all four beta diversity metrics.

**FIGURE 8:**
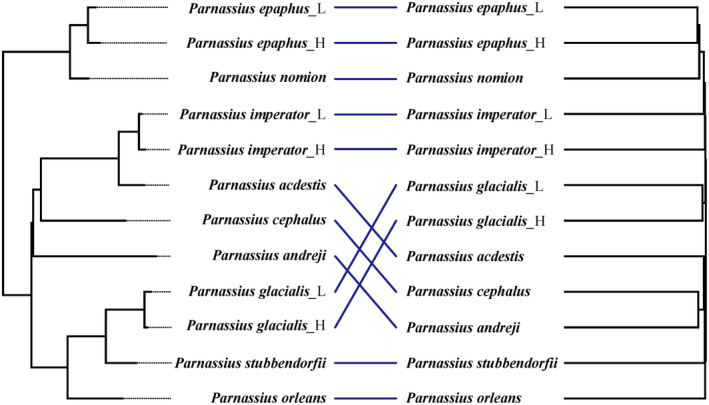
Co‐dendrogram of the gut bacterial communities and the host phylogeny. Left: the phylogenetic tree of 12 populations covering 9 *Parnassius* species based on the nuclear (37S_no_missing) dataset according to He et al. (2023); Right: the cluster dendrogram analyzed based on the gut bacterial communities using the Bray–Curtis metric.

We consistently found evidence in support of phylosymbiosis based on co‐dendrogram analysis, regardless of the method used for selecting representative microbial samples (i.e., randomly selected or population‐average gut microbial communities) or host phylogeny (i.e., mitochondria‐ or nuclear‐based tree). However, significantly higher congruence than expected by chance between the host phylogeny and microbial dendrogram was found only for beta‐diversity metrics of Jaccard and Bray–Curtis (*p* < .02, mean RF distance = 0.8, Figure 8, Table S7), but not for the weighted and unweighted UniFrac metrics, suggesting the intimate correlation between host phylogeny and gut microbial taxonomy related to recent microbial evolution.

**FIGURE 9:**
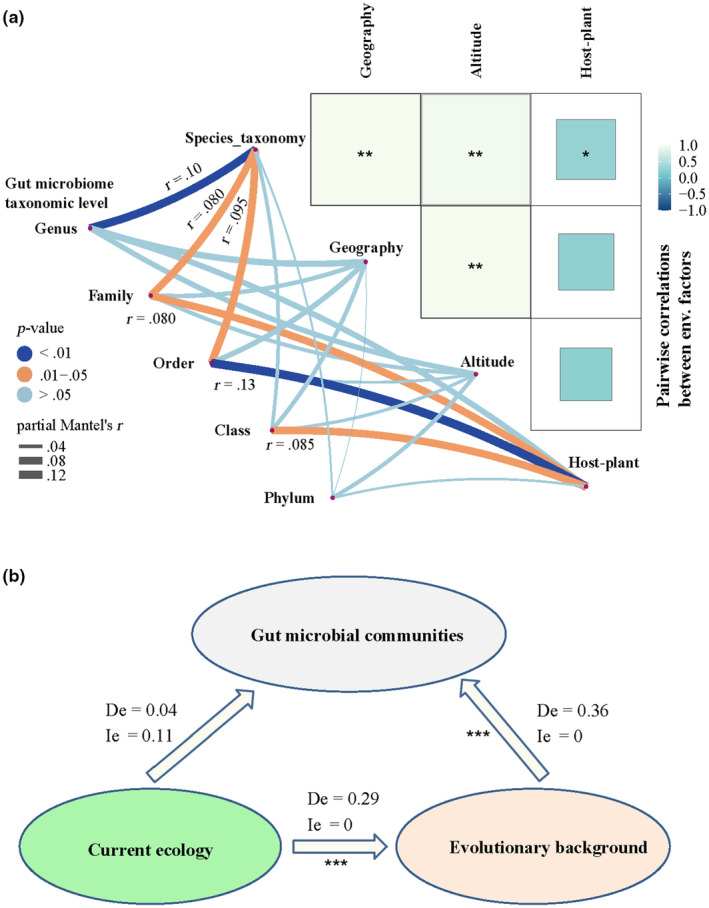
Correlations between the gut microbiome and potential factors. (a) Microbial composition at different taxonomic levels and potential influence factors. The color and width of the link line (left) between node and potentially influence factor indicate the significance and robustness of the correlation, respectively. For significant correlation (*p*‐value < .05), partial Mantel's *r* values were shown beside the link line. Microbial composition consists of the top 50 genera and families, 30 orders, 20 classes, and the top 10 phyla in relative abundance levels. The heatmap (right) shows the pairwise correlations between influence factors (**p‐value < .01; *p‐value < .05). (b) The direct and indirect effects of influence factors on gut microbial communities. Current ecological factors consist of altitude and sampling locality (based on the information of longitude and latitude for each sample), while host phylogeny and larval host pant for the evolutionary background. De and Ie indicate the direct effect and indirect effect of each variable, respectively (***p‐value < .001). Results based on the nucleotide dataset of 37S_no_missing for host phylogenetic distance matrix were displayed herein, similar to those based on other nucleotide datasets herein.

Moreover, host species taxonomy was shown to be the most significant factor in explaining the variation of gut microbial composition at the genus level (partial Mantel test, *r* = .10, *p* = .002, Figure 9a). Interestingly, larval host plant change was strongly correlated with the variations of gut microbial composition at relatively higher taxonomic levels (e.g., family, order, and class, partial Mantel test, *r* > .07, *p* < .05, Figure 9a). Given that microbial community variations at higher taxonomic level usually corresponded to deeper evolutionary time for the old microbial clades, the above results probably indicated again that older microbial clades had stronger associations with larval host plant than younger clades, consistent with the result of co‐dendrogram analysis. In addition, result of partial least squares path modeling (PLS‐PM) showed that adult *Parnassius* gut microbial communities were significantly shaped by factors related to host evolutionary background (total effect Te = 0.36 ± 0.056, *p* = 3.8e‐20, consisting of host phylogeny and larval host plant use), rather than those associated with current ecology (Te = 0.15 ± 0.025, *p* = .29, consisting of altitude and sampling locality) (Figure 9b). It is worthy to note that factors about current ecology were shown to significantly affect those of host evolutionary background (Te = 0.29 ± 0.050, *p* = 1.2e‐14), largely consistent with the result of heatmap above (Figure 9a) and that of the indirect effect (Ie = 0.11) on gut microbial communities for the current ecology (Figure 9b).

**FIGURE 10:**
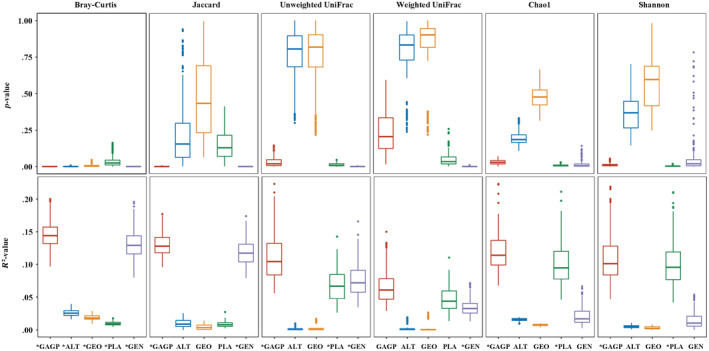
Host genetics and/or larval diet significantly explain the aspects of gut microbiome diversity, based on multiple regression on distance matrices (MRM) models using four beta‐diversity and two alpha‐diversity metrics. For each metric, the variance and *p*‐value explained by the full model with four factors (GAGP), then those explained by models with just altitude (ALT), geography (GEO), host plant (PLA), and genetics (GEN) were shown, respectively. The boxplots show the distribution of values obtained when running MRM on each of the 100 random dataset subsets. Asterisk denotes significance (*p* < .05 for most of dataset subsets). Box centerlines, edges, whiskers, and points signify the median, interquartile range (IQR), 1.5× IQR, and >1.5× IQR, respectively.

Based on 100 subsamples of the gut microbial dataset, analysis of relative contributions of geography, altitude, host genetics, and larval host plant to the microbial community structure with multiple regression on distance matrices (MRM) models showed that all four factors together explained 7.5%–23.3% of microbial community variations based on taxonomy‐based metrics (e.g., Jaccard and Bray–Curtis distances), and 2.3%–21.6% variations based on phylogenetically weighted metrics (e.g., unweighted UniFrac and weighted UniFrac distances) (Figure 10, Table S8). Analyses with different host distance matrices produced similar results, and showed that the effect of host genetics was largely significant, while models explained less variance for the phylogenetically weighted distances (Figure 10, Table S8). In contrast, larval host plant explained more observed variance for phylogenetically weighted distances than for taxonomy‐based distances (Figure 10), indicating again that there could exist phylogenetic correlation between host plant and gut microbes. Generally, both altitude and geography factors mostly had no significant effect on microbial community variation, although both contributed to the observed community variation based on the Bray–Curtis metric to some extent (Figure 10, Table S8). Our results also showed that larval diet (host plant), rather than the host genetics, was the significant explanatory variable for the alpha‐diversity variation (e.g., Chao1 and Shannon indices, *p* < .03, Figure 10, Table S8), regardless of phylogenetic distance matrices. Results of step regression containing all combinations of different variables showed that all four factors together explained the highest microbial diversity and community variations when compared to those from other combinations (Table S9), regardless of host genetic distance or microbial alpha‐ and beta‐diversity matrices used for analysis.

### 2.6 Biomarkers and the co‐occurrence of keystone microbial taxon in different *Parnassius* subgenera

**FIGURE 11:**
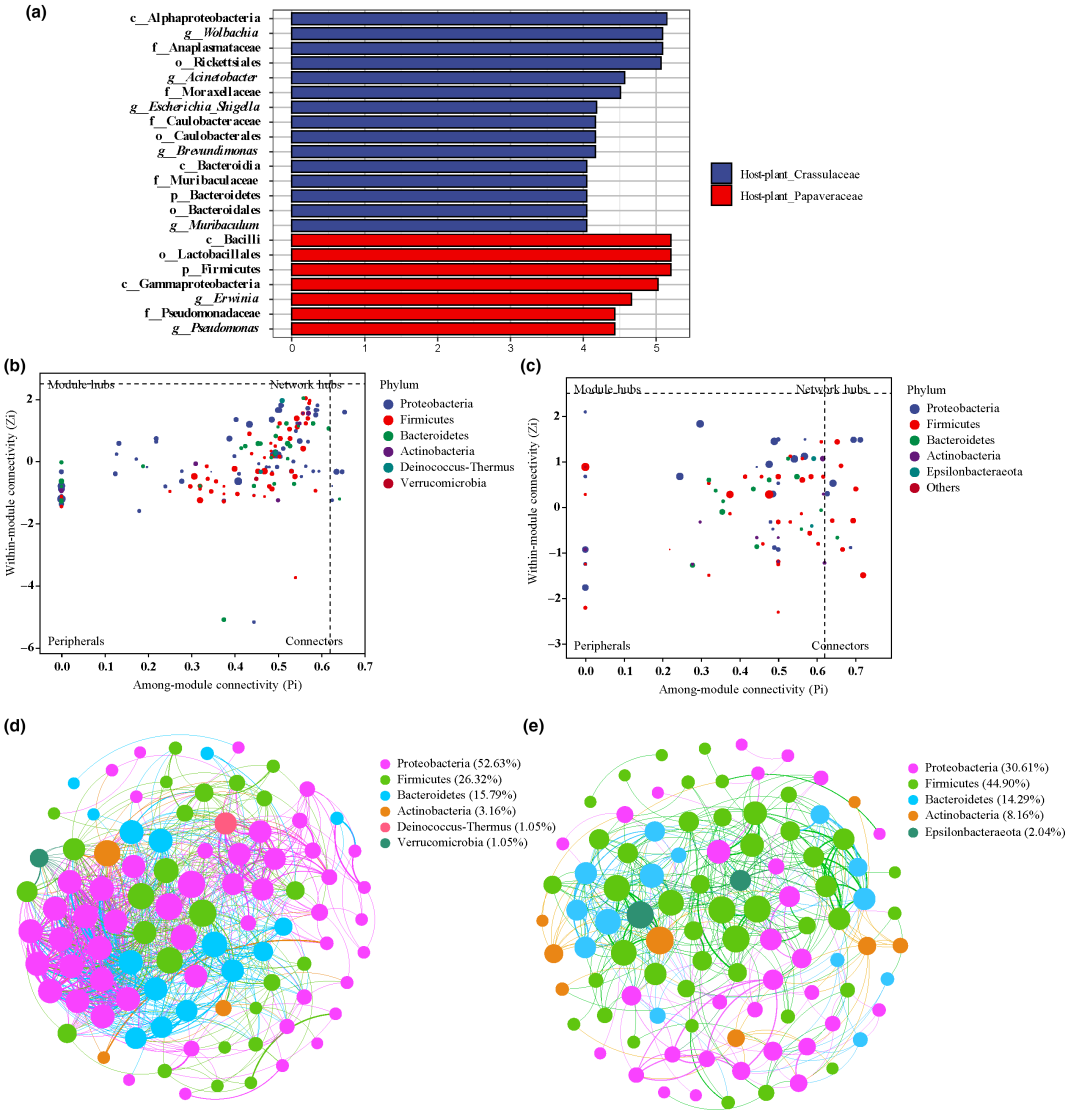
Biomarkers and the co‐occurrence of keystone microbial taxon for representative *Parnassius* species of the subgenus *Parnassius* (host plant: Crassulaceae + Saxifragaceae, left) and others (host plant: Papaveraceae, right), close to each other in altitude and region. (a) Biomarkers at different taxonomic levels were shown, based on LEfSe analysis (LDA > 4.0, *p* < .05). (b, c) ZP plots indicate the potentially keystone microbial species, according to the distribution of ASVs based on their module‐based topological roles. Each dot represents an ASV in the dataset of different phyla. The topological role of each ASV was determined according to the scatter plot of within‐module connectivity (Zi) and among‐module connectivity (Pi). (d, e) Co‐occurrence networks. Each node signifies an ASV, which could correspond to a microbial population. Colors of the nodes indicate different major phyla. With pair‐wise correlation of the ASVs, the network connection shows the robust (Pearson's *r* > .6) and significant (*p* < .05) correlation. The size of each node is proportional to the number of connections, and the width of link line between nodes corresponds to the correlation value.

Given that both host genetics and larval host plant could drive the significant variation of gut microbial composition for *Parnassius* species herein, the linear discriminant analysis (LDA) combined with effect size measurement (LEfSe) was performed to detect different biomarkers between the subgenus *Parnassius* (host plant: Crassulaceae + Saxifragaceae) and others (host plant: Papaveraceae). To alleviate potential bias resulting from the influence of different sampling locations, species (including *P. epaphus*, *P. mercurius*, *P. nomion*, and others in Menyuan and Qilian mountainous regions, Table S1) sampled at close geographic region and altitude were used for analysis. At the phylum level, the Bacteroidetes was specifically enriched in the subgenus *Parnassius* samples, while the Firmicutes enriched in samples of the other subgenera (Figure 11a). At the generic level, the microbial taxa *Wolbachia*, *Acinetobacter*, *Escherichia*, *Brevundimonas*, and *Muribaculum* were specifically enriched in the subgenus *Parnassius* samples, whereas the *Erwinia* and *Pseudomonas* were enriched for other *Parnassius* subgenera (Figure 11a). Among these bacteria genera, *Wolbachia* has been reported to not only colonize reproductive tissues of Lepidoptera, known for its role in sex ratio distortion, but also can increase the resistance of their hosts to pathogens, leading to increased longevity and fecundity of the lepidopteran host (reviewed in Duan et al., 2020; Liu & Guo, 2019; Paniagua Voirol et al., 2018); *Acinetobacter*, *Erwinia*, and *Pseudomonas* in insects could facilitate the degradation of host plant toxin (e.g., tea saponin, caffeine, and terpene), or cause soft rot, necroses, and wilt on a variety of plants, providing evidence that gut bacteria mediate adaptation of herbivorous insects to phytochemical resistance (Ceja‐Navarro et al., 2015; Chen et al., 2020; Li, Huang, et al., 2022; Starr & Chatterjee, 1972); and *Brevundimonas* had significant correlations with the concentrations of differential metabolites (e.g., phospholipids and certain amino acids) in insect hemolymph (Li, Zheng, et al., 2022). Metabolites produced from *Muribaculum* could contribute to the gut barrier integrity and support the defense against inflammation (reviewed by Sharma et al., 2021).

For the subgenus *Parnassius*, the co‐occurrence network analysis showed that vast majority of nodes were peripherals, representing the special microbiota in the microbial network. However, only six nodes (2.6%, 6/232) were connectors (similar to generalists present in majority of *Parnassius* species), which were derived from Proteobacteria and Bacteroidetes (Figure 11b). For the other subgenera of *Parnassius*, 15 nodes (13.8%, 15/109) were shown to be connectors, which were derived from the Firmicutes, Proteobacteria, Bacteroidetes, and Actinobacteria (Figure 11c). Proteobacteria was the most co‐occurrence phylum (52.63%) for the subgenus *Parnassius* (Figure 11d), while the Firmicutes was the most (44.90%) for the other subgenera (Figure 11e). In addition, among the top 10 genera with high co‐occurrence (Figure S3, including *Wolbachia*, *Saccharibacter*, *Serratia*, *Brevundimonas*, *Escherichia*, *Acinetobacter*, *Carnobacterium*, *Erwinia*, *Enterococcus*, *Pseudomonas*, *Megamonas*, etc.), only 1 genus (*Serratia*) was shared between the two *Parnassius* groups above. Moreover, both *Serratia* and *Megamonas* were shown to be connectors but only found in other subgenera of *Parnassius* (Figure S3). Most of these bacteria genera contribute to digestion, detoxification, or defense against pathogens, indicating the adaptations to different insect hosts, altitudes, and host plants on the QTP over long evolutionary time. For example, *Saccharibacter*‐related microbes have the ability to degrade cellulose, hemicellulose, and pectin (Starr et al., 2018); *Serratia* can promote the metabolism of wood materials and decrease toxic compounds (e.g., monoterpenes and diterpene acids) present in plant (Adams et al., 2013; Boone et al., 2013), and also can improve aphid fitness by disrupting the predation strategy of ladybeetle larvae (Wang et al., 2024); *Acinetobacter* was found to have lignin degradation activity in previous experiments (Ghodake et al., 2009); *Megamonas*, a free hydrogen utilizer and short‐chain fatty acid producer, has been shown to inhibit *Salmonella* growth in vitro (Sergeant et al., 2014); and *Enterococcus* and *Carnobacterium* are likely responsible for lignocellulose and carbohydrates digestion for insect hosts, respectively (Anand et al., 2010; Li et al., 2017).

However, some common gut bacterial inhabitants may be detrimental or beneficial depending on the community composition of the host gut (Paniagua Voirol et al., 2018). On the other hand, one keystone microbial symbiont (e.g., *Bacillus subtilis* or *Pseudomonas aeruginosa*) can alter the gut microbiota community structure and insect health (Li, Zheng, et al., 2022; Zhang et al., 2021). Shifts in the relative abundance of *Wolbachia*, *Enterococcus*, *Pseudomonas*, and *Brevundimonas* have been shown to significantly influence the concentrations of differential metabolites (e.g., phospholipids and certain amino acids) in insects, which have a diverse range of functions (Duan et al., 2020; Li, Zheng, et al., 2022; Liu & Guo, 2019; Zhang et al., 2021). These previous findings have provided concrete evidence that alterations in gut microbiota could drive the variation in circulating metabolites and physiological state of insect hosts (Li, Zheng, et al., 2022). Together, considering the important functions and co‐occurrence for biomarkers or keystone microbial taxa, both the different biomarkers and alteration of co‐occurrence network pattern between two *Parnassius* groups above probably contributed to the variation in circulating metabolites and physiological state of hosts, reflecting a potential adaptive mechanism of butterfly hosts in response to harsh environment on the QTP.

### 2.7 Potential functions of gut microbiota for papilionid butterfly species

Results of KEGG functional prediction for the gut microbiota based on phylogenetic investigation of communities by reconstruction of unobserved states 2 (PICRUSt2) consistently showed that metabolism‐related categories (KEGG level 2, including those of carbohydrate, amino acid, cofactors, vitamins, terpenoids, polyketides, etc.) harbored higher level of abundance among all KEGG categories, reflecting the potentially active metabolic functions of gut microbiota for the butterfly species of this study (Table S10, Figure S4). Moreover, significant differences in relative abundance level for the metabolism category (KEGG level 1, Wilcoxon rank‐sum test, *p* < .001) were detected between *Parnassius* (including HA and LA groups, mean ± SD: 78.1 ± 3.9%) and other two papilionid species (OG: 82.1 ± 0.46%).

Furthermore, according to the prediction results based on the MetaCyc database, the biosynthesis category (MetaCyc level 1, including amino acid, cofactor, electron carrier, vitamin, nucleotide biosynthesis, etc.) was found to harbor the highest abundance level (HA and LA: 68.0 ± 7.3%; OG: 63.4 ± 5.7%) and constituted the top three functional categories in terms of abundance level (Table S11, Figure S5) along with other two categories related to degradation and generation of precursor metabolite and energy (MetaCyc level 1).

## 3 DISCUSSION

Understanding the factors that shape gut microbial communities in insects on the QTP is of great interest. Here, we sampled the *Parnassius* butterfly species which utilize different plants as the larval diet and inhabit variable high mountain areas as the complex symbiotic system to quantify the relative contributions of host genetics, geographic distance, elevation, and larval diet to the gut microbial characteristics under the extremely severe conditions. We, firstly, found that gut microbiomes are generally similar in both alpha and beta diversity among intraspecific individuals, regardless of sampling time and locality, and secondly, detected the strong effect of both host genetics and larval diet on the gut microbial diversity and symbiotic community structure. In addition, our results also suggested that host genetics and larval diet were contributed to the different aspects of gut microbial diversity and that gut microbes associated with larval diet were likely to acquire at deeper evolutionary time scales than those associated with more recent host speciation events, as demonstrated in mammalian taxa (Donohue et al., 2022).

### 3.1 Host genetics mainly modulated gut microbial beta diversity, supporting phylogenetically structured adult‐stage microbiomes and intimate host–microbe interactions for *Parnassius* species

Based on results of PERMANOVAs, partial Mantel tests, and MRMs, host genetics was generally proved to be a strong predictor of microbial beta diversity related to the symbiotic community structure, regardless of phylogenetic distance matrices. We consistently found evidence of phylosymbiosis using both co‐dendrogram and correlation‐based analyses, although herein more congruence was shown for taxonomy‐based metrics (e.g., Jaccard and Bray–Curtis distances) than for phylogenetically weighted metrics (e.g., unweighted UniFrac and weighted UniFrac distances). Both contemporary host‐filtering environmental microbes and host–microbe coevolution can contribute to phylosymbiosis (Donohue et al., 2022; Hammer et al., 2020). In the present study, results of the partial Mantel tests and MRMs consistently showed that larval host plant explained more observed microbial variance based on phylogenetically weighted metrics than taxonomy‐based metrics, indicating phylogenetic correlation between host plant and gut microbes, especially those in old microbial clades. Actually, similar environments for larvae and adults, and species dispersal limitations that can restrict the species range can be found for *Parnassius* species in high‐altitude mountainous regions according to our field work. For the same species at larval and adult stages, similar sources of microbiota related to host plants could exist and be filtered by hosts. Therefore, the phylogenetic structure in *Parnassius* microbiomes could arise from the larval diet (one conserved host trait) that differentially filters microbes from the environment, and physiological variation among the hosts as suggested by the results of biomarkers and the co‐occurrence network pattern analysis. Given the heterogeneous local environments and significant differences in soil bacterial communities of different sites across alpine ecosystems on the QTP and other regions (Li et al., 2023; Wang et al., 2023), we suggested that adaptive host filtering associated with specific host trait (e.g., larval host plant use) was closely associated with host–microbe interactions, jointly contributing to the phylosymbiosis of *Parnassius* species.

Vertical transmission across generations, strong host selectivity, and fitness dependencies can jointly contribute to host–microbe coevolution (Groussin et al., 2020). For lepidopteran species, due to the physiological changes during metamorphosis and the diet variations, considerable change in gut microbial community from larvae to adults has been reported (reviewed in Paniagua Voirol et al., 2018). However, some core bacteria persist in holometabolous insects including Lepidoptera, probably through vertical transmission. For example, persistent maternal transmission of bacterial endosymbionts in lepidopterans has been demonstrated for two symbionts (*Wolbachia* and *Spiroplasma*) (Ahmed et al., 2015; Jiggins et al., 2000; Narita et al., 2007); caterpillar of large cabbage white butterfly *Pieris brassicae* feeding on egg shell can take up symbionts maternally transferred via the egg (Salem et al., 2015). Similarly, transgenerational immune priming by means of maternal transfer of bacteria or bacteria‐associated compounds has also been observed in the moth *Trichoplusia ni* (Freitak et al., 2009). Vertical transmission of microbial symbionts with distinct routes has also been reported from other insect groups (Bistolas et al., 2014; Hosokawa & Fukatsu, 2020; Khan et al., 2020; Mallott & Amato, 2021; Wu et al., 2023). It has been reported that microbiomes of pollen‐feeding *Heliconius* species were not uniquely distinct from those of nonpollen‐feeding ones, suggesting diet at the adult stage (unique to *Heliconius* butterflies) is not a primary driver of the gut microbiome (Hammer et al., 2020). Moreover, previous study also suggested that only few butterfly species showed significant dietary and developmental transitions in bacterial communities, suggesting weak impacts of dietary transitions across the development of the same species (Phalnikar et al., 2018). To date, how Lepidoptera gains and retains gut bacterial members is a largely unresolved question. For the *Parnassius* species herein, gut microbes may be horizontally transmitted through the host plant or environment near the plant but also can be vertically via the egg stage. However, evidence for vertical transmission of gut symbionts is still scarce and needs further investigations.

On the other hand, the dynamic bi‐directional interactions between mitochondria and the gut microbiome provided by reactive oxygen species (ROS) have also been systematically reviewed (Ballard & Towarnicki, 2020), suggesting the reciprocal selection between mitochondria and the gut microbiota over the evolutionary timescale. Moreover, given that nuclear genes mediating innate immunity are linked to gut microbial variation in model organisms (Kurilshikov et al., 2017), similar mechanism might also contribute to nuclear gene selection on the gut microbiota primarily mediated by host genetics. These results consistently indicated that there could exist signals of the strong host selectivity implying the host–microbe coevolution in *Parnassius* species.

Additionally, our results showed that host genetics controlled not only gut microbial composition but also the stability of these communities across intraspecific *Parnassius* populations sampled at different times and at different localities (hundreds to thousands of kilometers away). Actually, besides the impact on digestion, nutrient acquisition, and protection against pathogens by gut microbiota, symbiont‐induced behavioral alterations, in particular those regarding mating behaviors, may potentially lead to reproductive isolation and speciation of the host insects (Hosokawa & Fukatsu, 2020). Our results of biomarkers, co‐occurrence networks, and potential function prediction (e.g., biosynthesis of amino acid, cofactor, vitamin, and nucleotide; degradation and generation of precursor metabolite and energy; etc.) supported that gut microbes appeared to be highly co‐adapted with host species and to have evolved as mutualists for millions of years, to overcome the extremely severe environments on the QTP. The stability of gut microbial composition across life stages was also reported for most of 12 butterfly species across different developmental stages and intraspecific populations in other insect groups (Hammer et al., 2014; Phalnikar et al., 2018; Shukla & Beran, 2020), and for particular example, although the plant leaf metabolites influenced the plant microbiota, this effect did not impose on microbiota of the *Melitaea cinxia* caterpillars feeding on those leaves (Minard et al., 2019). The above evidence consistently suggested that insect gut microbial communities were phylogenetically conserved in their respective host species, indicating strong host–microbe fitness dependencies. Together, results herein supply the signal of coevolution between *Parnassius* species and gut microbes, which could have occurred over long evolutionary timescales, as found previously in other insects (Groussin et al., 2020; Khan et al., 2020; Koch et al., 2013; Koskella & Bergelson, 2020; Sloan & Moran, 2012) and plants (Abdelfattah et al., 2022; Mesny et al., 2023).

### 3.2 Larval host plant functionally influenced gut microbiome evolution, underlying intimate insect–microbe–plant interactions and high‐altitude adaptations

In the present study, we discovered that the dominant microbes are largely shared among *Parnassius* species at the generic level, although the relative abundance for each was generally species and population specific. Among the dominant genera of the top 20 relative abundance, at least 7 genera (e.g., *Enterococcus*, *Serratia*, *Saccharibacter*, *Pseudomonas*, *Erwinia*, *Enterobacter*, and *Acinetobacter*) can help insects to degrade cellulose, hemicellulose, lignin, pectin, monoterpene, or diterpene acids, decreasing the toxic compounds present in plant (Adams & Boopathy, 2005; Anand et al., 2010; Berman et al., 2018; Chen et al., 2020; Kim et al., 2017; Li et al., 2017); while other dominant genera, including *Carnobacterium*, *Weissella*, *Wolbachia*, and *Rickettsiella*, have been found to be involved in the metabolism of nutrients for the function of promoting resistance to insect pathogens and increasing survival rate (Anand et al., 2010; Khan et al., 2020; Liu & Guo, 2019). These results supported the hypothesis that bacteria actively colonized the butterflies, and preliminarily domesticated these microbes when *Parnassius* larvae fed on the Crassulaceae or Papaveraceae plants over long evolutionary timescale. We also found that about 10 of the top 20 microbial genera herein are generally shared with those in other adult insect groups including butterflies, moths, honey bees, and sawyer pine beetles (Chen et al., 2020; Hammer et al., 2020; Khan et al., 2020; Li et al., 2017), probably suggesting the gut microbial composition of field‐collected insect species are of good stability and specificity. Although natural diets (e.g., nectar and pollen) for the butterfly at adult stage were different from those at larval stage (e.g., leaves of the host plant), the dominant genera of gut microbiota in the adult *Parnassius* butterfly could be stably maintained at proper levels. Such storage‐like effects associated with larval diet could play key roles in maintaining diverse microbial communities for the subsequent adult‐stage (Bistolas et al., 2014) and in maintaining diverse symbionts that allow adult hosts to recover from perturbation, promote dispersion, and acclimate to heterogeneous environments in *Parnassius* (Martens et al., 2014).

Moreover, Mantel tests and MRM analyses all indicated that larval host plant was significantly correlated with both the alpha and beta diversity of the gut microbiomes in adult *Parnassius* species. It is worthy to note that, higher correlation values of host plant on microbial beta diversity were found based on UniFrac metrics, but not Jaccard and Bray–Curtis metrics. Both the Jaccard and Bray–Curtis metrics used microbial taxonomy to measure microbial community structure, making these metrics more sensitive to recent microbial evolution. In contrast, both weighted and unweighted UniFrac metrics are phylogenetic measures of diversity, as microbial clades with longer branch lengths (i.e., deeper evolutionary history) are weighted (Donohue et al., 2022; Sanders et al., 2014). Therefore, combined with the results of partial Mantel tests and MRM analyses, we suggested that larval host plants influenced the structure of ancient microbial clades, but had no significantly discernable effects on recently evolved microbial clades. Host plants at the generic level for *Parnassius* butterfly species, including *Corydalis* (Papaveraceae), *Sedum*, *Rhodiola*, *Pseudosedum* (Crassulaceae), as well as *Saxifraga* (Saxifragaceae), originated and colonized the QTP region at about 37–21 million years ago (Ma) in the late Oligocene to early Miocene (Ebersbach et al., 2017; Perez‐Gutierrez et al., 2015; Zhang et al., 2014). Thus, host plants' rapid diversification was shown to slightly predate the divergence between the subgenus *Parnassius* (feeding on Crassulaceae + Saxifragaceae) and other subgenera (feeding on Papaveraceae) (Condamine et al., 2018; Su et al., 2020), necessary for the successive adaptive radiation of *Parnassius* species. Taken together, considering the fundamental roles (e.g., digestion, detoxification, protection against pathogens, even reproductive manipulation, etc.) of the gut microbiomes in lepidopterans (Paniagua Voirol et al., 2018), multiple lines of evidence supported that certain microbial clades associated with larval host plant could functionally colonize the gut before the *Parnassius* host's adaptive radiations.

Since Ehrlich and Raven proposed the insect–plant interactions driven by diffuse coevolution over long evolutionary periods (Ehrlich & Raven, 1964), numerous cases of butterfly–plant coevolution have been found, such as the furanocoumarin‐feeding *Papilio* (Papilionidae), glucosinolate‐feeding Pierinae (Pieridae), and others (Edger et al., 2015; Nylin & Wahlberg, 2008; Wheat et al., 2007). Particularly, convergent resistance to GABA receptor neurotoxins through insect–plant coevolution has been found recently (Guo et al., 2023). Moreover, evidence has also been found for host–microbiome coevolution in plant groups (Abdelfattah et al., 2022). For the *Parnassius* butterfly group, genome‐wide coevolution between host species and their larval host plants, and their adaptive radiation was attributed to the historical host plant shifts that have been reported (Allio et al., 2021). Our study showed that host genetics modulated the beta diversity and community structure of gut microbiomes, and host plant mainly influenced the function and evolution of gut microbiomes, providing a complete chain of evidence for the intimate insect–microbe–plant interactions in *Parnassius* butterflies.

The intimate insect–microbe–plant interactions also can provide a functional clue for the high‐altitude adaptations. Complex insect–microbe and intermicrobial interactions could be selected during evolution, promoting the survival and fitness of insects and their associated microbes (Mesny et al., 2023). In the severe alpine environment of QTP, the relatively stable relationship among mutualists is beneficial for the butterfly host to acquire sufficient nutrients, maintain energy balance, and then maintain vitality and reproductive ability. On the other hand, host insects can provide a relatively stable endogenous environment for the gut microbiota, promoting their survival and potential vertical transmission. Concurrently, under the selective pressure of environments on the QTP (e.g., high ultraviolet radiation), the accelerated evolutionary rate for certain microbial taxa could be detected, increasing their evolutionary probability of digesting new metabolic components in the process of host plant shift, and thus contributing to the adaptive radiation of *Parnassius*. However, more detailed studies on insect groups are warranted to validate this hypothesis in future.

## 4 CONCLUSIONS

Based on the aggregated genome‐based phylogeny and microbial data of *Parnassius* species at the population level which were generated with the same collection methods, we utilized a robust analytical framework to resolve the relative importance of host genetics, geography, altitude, and larval host plant on gut microbial community structure and evolution. Our findings indicate that both host genetics and larval host plant modulated gut microbial diversity and community structure. Moreover, we decoupled the effects of host genetics and larval diet on gut microbiome and showed that host genetics mainly governed gut microbial beta diversity and community structure, while larval host plant functionally influenced gut microbiome evolution. Results above jointly support the hypothesis of the coevolution between *Parnassius* species and gut microbes over long evolutionary timescales, providing a clue for the underlying insect–microbe–plant interactions and high‐altitude adaptation mechanisms. Our findings help to resolve the major modulators of gut microbiome diversity and evolution in butterfly species, which have not been well studied in wild insects with varying degrees of ecological niche divergence and evolutionary relatedness, especially those inhabiting the QTP.

## 5 MATERIALS AND METHODS

### 5.1 Sample collection

All imago individuals (*n* = 225) from 16 populations, representing 13 papilionid butterfly species at 12 sites were field collected during the daytime between 10:00 a.m. and 1:00 p.m., mostly in July 2020 and 2021 (Table S1). These sites spanned more than 2000 km transect from northwestern to southeastern China and included subalpine meadows, alpine arid desert habitats, as well as subtropical warm humid habitats (Figure 1). With the exception of *P. glacialis*, *Sericinus montela*, and *Papilio polytes*, all *Parnassius* samples were collected from the QTP region, mainly covering the range of Qilian Mountain in China (Table S1, Figure 1). According to the sampling locality and altitude distribution pattern, samples here were divided into three main groups, classified as the high‐altitude group (HA, >2800 m above sea level, a.s.l.), low‐altitude group (LA, mostly 200–1800 m a.s.l. for *P. glacialis*), and other group (OG, mostly in plain area for *S. montela* and *P. polytes*), respectively (Table S1).

All samples were initially preserved in absolute ethyl alcohol in the field and transferred to −80°C until DNA extraction. Midguts of imago samples for each species were dissected under sterile conditions, with slight modifications according to the previous study (Cao et al., 2021). In brief, after washing samples three times with 75% ethanol for 90 s followed by three rinses with ddH_2_O for 90 s, all individuals for each species were dissected in physiological saline. In total, 75 samples in 16 populations were dissected herein, and each sample contained three individual midguts.

### 5.2 DNA extraction, PCR amplification, and Illumina sequencing

The gut digesta samples (~200 mg of each sample) were used for microbial DNA extraction using DNeasy PowerSoil Kit (Qiagen, Netherlands), following manufacturer protocols. Total DNA samples were assessed by 0.8% agarose gel electrophoresis and quantified using NanoDrop ND‐1000 spectrophotometer (Thermo Fisher Scientific, Waltham, USA). The V3–V4 region of the 16S rRNA gene was amplified using the primer pair 338F/806R (5′‐ACTCCTACGGGAGGCAGCA‐3′ and 5′‐GGACTACHVGGGTWTCTAAT‐3′). Amplicons were generated in 25 μL volumes using Phusion Hi‐Fidelity Polymerase (TransGen Biotech, Beijing, China) containing 0.4 μM of forward and reverse primers and 40 ng of template DNA. Reaction conditions for 16S rRNA gene amplification were as follows: 98°C 2 min, 30 cycles of 98°C for 15 s, 55°C for 30 s, and 72°C for 30 s, followed by a final extension of 72°C for 5 min.

The PCR products were extracted from 2% agarose gel and further purified using VAHTS DNA Clean Beads (Vazyme, Jiangsu, China) and quantified with the PicoGreen dsDNA Assay Kit (Invitrogen, Carlsbad, USA) and FLx800 Fluorescence Microplate Reader (BioTek, Winooski, USA). Afterward, sequencing libraries were constructed using TruSeq Nano DNA LT Library Prep Kit (Illumina, San Diego, CA, USA), and paired‐end sequencing was performed on the Illumina NovaSeq PE250 platform (Personal Biotech, Shanghai, China).

### 5.3 Bioinformatic processing of the sequence data and identification of microbial taxa

Illumina sequencing reads were filtered and processed using QIIME2 v. 2019.4 (Quantitative Insights into Microbial Ecology 2; Bolyen et al., 2019). Briefly, raw sequence data were demultiplexed using the demux plugin followed by primers cutting with cutadapt v2.3 (Martin, 2011), and then nonsingleton amplicon sequence variants (ASVs) were obtained after quality filtered, denoised, merged, and chimera removed using the DADA2 plugin (Callahan et al., 2016), and chloroplastic, mitochondrial, and nonbacterial ASVs were also removed. Classification of ASVs at various taxonomic levels was conducted by the classify sklearn naive Bayes taxonomy classifier in feature classifier plugin against the Silva reference database (Release 138; Quast et al., 2013). A phylogeny was inferred for all ASV sequences with fasttree based on a multiple‐sequence alignment generated by mafft (Katoh & Standley, 2013; Price et al., 2010).

### 5.4 Estimation and comparison of microbial alpha and beta diversity and the influential factors

To reduce the impact of differential sequencing depths, samples were rarified to an even depth (35,701 reads herein) according to the official tutorials (https://docs.qiime2.org/2019.4/tutorials/). Both the alpha diversity (including indices of observed species, Chao1, Shannon, and Simpson index) and beta diversity (based on Jaccard, Bray–Curtis, weighted UniFrac, and unweighted UniFrac metrics) among groups were compared on the genescloud platform (www.genescloud.cn, Personal Biotech, Shanghai, China). The Jaccard metric only accounts for the presence/absence of ASVs, while the Bray–Curtis additionally accounts for differences in the ASVs' abundance, both using microbial taxonomy (not phylogeny) to measure microbiome community, sensitive to recent microbial evolution. Compared to the Jaccard and Bray–Curtis metrics, both weighted UniFrac and unweighted UniFrac are phylogenetic measures of diversity, and can effectively minimize the influence of recent microbial evolution in community divergence, as microbial clades with longer branch lengths (i.e., deeper evolutionary history) are weighted. Furthermore, weighted UniFrac also incorporates both the presence/absence and abundance of ASVs, while unweighted UniFrac only consider presence/absence of ASVs. Therefore, all four beta‐diversity metrics above have known strengths and weaknesses for analyses, and leveraging these differences can help identify drivers of microbial community divergence (Sanders et al., 2014). In addition, the Good's coverage index was used to characterize the sequencing depth for samples. After initial examining taxonomy and clustering patterns based on alpha‐diversity analysis and DCA (detrended correspondence analysis), eight outlier samples were excluded from subsequent analyses (Table S1).

Principal coordinate analysis (PCoA) and nonmetric multidimensional scaling analysis (NMDS) accompanied by the permutational multivariate analysis of variance (PERMANOVA, permutations = 999) were conducted on the genescloud platform to assess the significance of microbial community differences among groups (McArdle & Anderson, 2001). Results based on Bray–Curtis and/or Jaccard metrics were displayed, even for those substantially different from other metrics.

To examine how host genetics, geography, altitude, and host plant independently influenced microbial composition and further to test for phylosymbiosis in *Parnassius* butterfly species, partial Mantel tests with the Spearman method and 999 permutations were conducted using *ecodist* v2.0.9 (Goslee & Urban, 2007). Environmental variables were converted to distance matrices through various means as performed previously (Youngblut et al., 2019). Host phylogenetic distance matrices were generated using the Kimura 2‐parameter model with a gamma distribution (shape parameter = 3) in MEGA X (Kumar et al., 2018), based on three nucleotide datasets: (1) 37S_missing_0.25 (5,532,419 autosomal single‐nucleotide variants [SNVs]); (2) 37S_no_missing (728,820 SNVs); and (3) 13 mitochondrial protein‐coding genes (13 PCGs, 10,347 bp), all of which were derived from the newly sequenced data of 37 samples in 12 populations covering 9 *Parnassius* species from our laboratory (Table S12) (He et al., 2023). The pairwise geographic distance (the great circle route) matrix was calculated using *geosphere* v1.5‐18 (Hijmans, 2022), based on the information of longitude and latitude for each sample. The host plant and altitude data were converted to the respective Gower distance.

The “co‐dendrogram” approach was also used to evaluate congruence between host phylogeny and microbiome dendrogram (also known as phylosymbiosis) using the phytools “cophylo” command with rotation (Revell, 2012). Specifically, we constructed unweighted pair group method with arithmetic mean (UPGMA) dendrograms for each beta‐diversity metric by both (1) calculating the mean relative abundance of each ASV across samples within a given population and (2) random picking one sample per population. Random picking was repeated 100 times per population to assess the intraspecies heterogeneity in microbiome diversity and the robustness of co‐dendrogram analysis. To obtain the host phylogeny, we used IQ‐TREE trees based on nuclear (37S_no_missing) and mitochondrial (13 PCGs) concatenated datasets (He et al., 2023), which has been pruned to include only the same or geographically close population for the same species in this study. Significance of topological congruence was calculated by comparing the normalized Robinson–Foulds (RF) distance to those from 10,000 randomized microbiome trees using the “rtree” function in ape (Paradis & Schliep, 2019).

Moreover, in order to explore the correlations between the gut microbiome at different taxonomic levels and potential factors, microbial composition data of samples (including the top 50 genera and families, 30 orders, 20 classes, and the top 10 phyla in relative abundance levels) and factors (e.g., species taxonomy, altitude, host plant use, and geography) were used to perform the partial Mantel test on the genescloud platform (www.genescloud.cn). Furthermore, partial least squares path modeling (PLS‐PM) based on the R package plspm (Sanchez et al., 2023) was used to assess the direct and indirect effects of latent variables (e.g., host evolutionary background and current ecology), which can be measured by multiple factors (manifest variables, including host phylogenetic distance, altitude, host plant use, and sampling locality), and to quantify the fractioning contributions of different factors to the gut microbial communities.

In addition, multiple regression on distance matrices (MRMs) were performed with the *ecodist* v2.0.9 R package to examine the relative contributions of each factor to both the alpha and beta diversity. All regression variables were converted to distance matrices as performed in partial Mantel tests. In addition, alpha diversity (represented by Chao1 and Shannon indices) was converted to the Euclidean distance matrices derived from all pairwise sample comparisons. Considering the gut microbial dataset mostly contained four or five samples per population, while nearly all the host nucleotide dataset included three samples per population, we conducted the sensitivity analysis to assess the intraspecies heterogeneity in microbiome diversity and host metadata. Therefore, 100 subsamples of the gut microbial dataset were generated, each with just three randomly selected microbial samples (except one population BQ_H, four samples) per host population (Table S12), to assess the sensitivity and robustness of partial Mantel test and MRM in this study. Furthermore, MRM step regressions containing all combination of different variables based on one random subsample of the gut microbial dataset were also conducted.

### 5.5 Biomarker and co‐occurrence network analysis

To find biomarkers that differed significantly among different groups, the linear discriminant analysis (LDA) combined effect size measurement (LEfSe) was used (LDA > 4.0, *p* < .05) in pairwise comparisons (Segata et al., 2011). The network analysis of microbial communities was conducted at ASV level to explore the co‐occurrence among microbes and identify the potential keystone taxa using the genescloud tools (https://www.genescloud.cn). Briefly, ASVs with total occurrences less than five across samples were filtered out, and then dominant species network of nodes (ASVs) with the top 100 mean abundance was built (Pearson's *r* > .6 and *p* < .05), through the SparCC algorithm and random matrix theory (RMT)‐based approach based on the abundance data of the remaining ASVs (Deng et al., 2012; Friedman & Alm, 2012). Afterward, the Zi and Pi values were calculated for each node in the network, referring to within‐module connectivity and among‐module connectivity, respectively. Finally, according to the Zi and Pi values, the role of each node in the network was determined, including peripherals, connectors, module hubs, and network hubs (Deng et al., 2012). The network was visualized using Gephi 0.10.1 (Bastian et al., 2009).

### 5.6 Potential function prediction

Based on the Kyoto encyclopedia of genes and genomes (KEGG, https://www.genome.jp/kegg/) and MetaCyc (http://metacyc.org/) databases, PICRUSt2 (phylogenetic investigation of communities by reconstruction of unobserved states 2) was used to predict functions of the gut microbiomes (Douglas et al., 2020; Langille et al., 2013), and then the normalized abundance values of each functional pathway were obtained to further explore the potential differences of gut microbiota among papilionid butterfly groups at the functional level.

## AUTHOR CONTRIBUTIONS

**Chengyong Su:** Conceptualization (lead); data curation (equal); formal analysis (lead); investigation (equal); writing – original draft (lead); writing – review and editing (lead). **Tingting Xie:** Formal analysis (equal); validation (equal); writing – original draft (equal); writing – review and editing (equal). **Lijun Jiang:** Data curation (lead); formal analysis (equal); methodology (equal); resources (equal); validation (equal); writing – original draft (equal). **Yunliang Wang:** Formal analysis (supporting); writing – original draft (supporting). **Ying Wang:** Formal analysis (supporting); investigation (equal); resources (equal). **Ruie Nie:** Formal analysis (equal); writing – original draft (supporting); writing – review and editing (supporting). **Youjie Zhao:** Formal analysis (equal); investigation (equal); resources (equal); writing – original draft (equal). **Bo He:** Formal analysis (supporting); resources (lead); writing – original draft (supporting). **Junye Ma:** Data curation (supporting); investigation (equal); methodology (equal); resources (equal). **Qun Yang:** Conceptualization (equal); data curation (supporting); funding acquisition (lead); methodology (equal); project administration (equal); resources (equal). **Jiasheng Hao:** Conceptualization (equal); data curation (supporting); funding acquisition (lead); methodology (supporting); project administration (lead); resources (equal); validation (equal); writing – original draft (equal); writing – review and editing (equal).

## FUNDING INFORMATION

This work received financial support from the CAS Strategic Priority Research Program to Q.Y. (Grant No. XDB26010204), the National Science Foundation of China to J.H. (Grant No. 41972029), and the Natural Science Foundation of Universities of Anhui Province to C.S. (Grant No. KJ2021A0100).

## CONFLICT OF INTEREST STATEMENT

The authors declare no competing interests for the publication of this study.

## Supporting information


Table S1.

Figure S1.

## Data Availability

Sequencing data are available in the NCBI Sequence Read Archive (BioProject PRJNA1018097). Metadata to the individual sequence samples are provided in Tables. Scripts and codes for QIIME2 and R are available at https://doi.org/10.6084/m9.figshare.25184525.v1.
