# Supplementary material for: Host genetics and larval host plant modulate microbiome structure and evolution underlying the intimate insect–microbe–plant interactions in Parnassius species on the Qinghai‐Tibet Plateau
Source: Ecol Evol. 2024 Apr 10;14(4):e11218. doi: 10.1002/ece3.11218 (PMC11007261; doi:10.1002/ece3.11218)
Supplement: Supplementary file 2 — Figure S1. [file ECE3-14-e11218-s001.pdf]

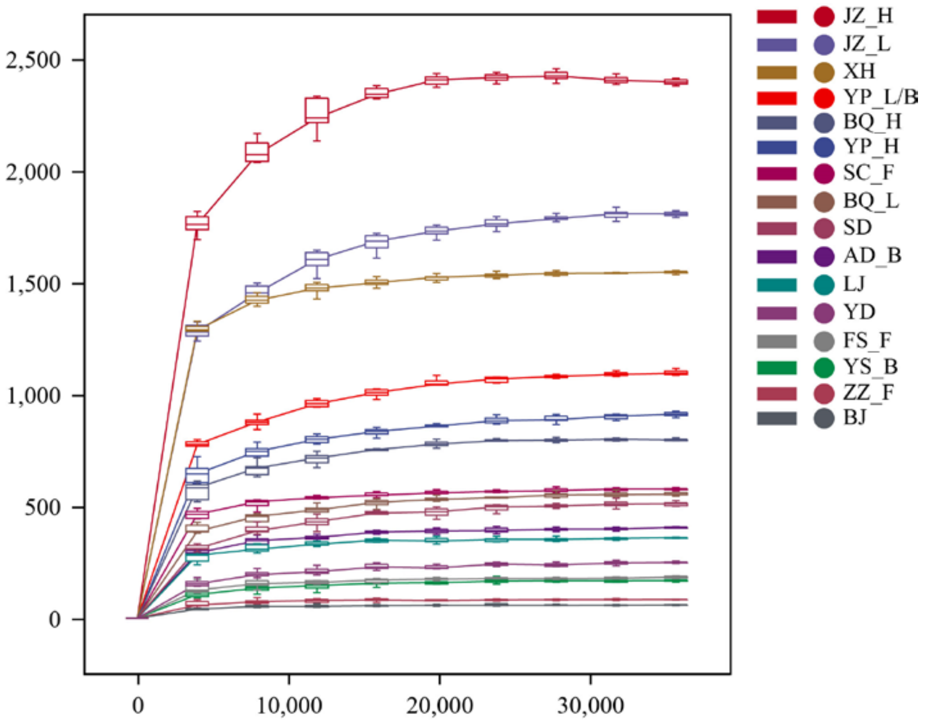

Figure S1 Rarefaction curve for each population indicating the sufficient sequencing depth.

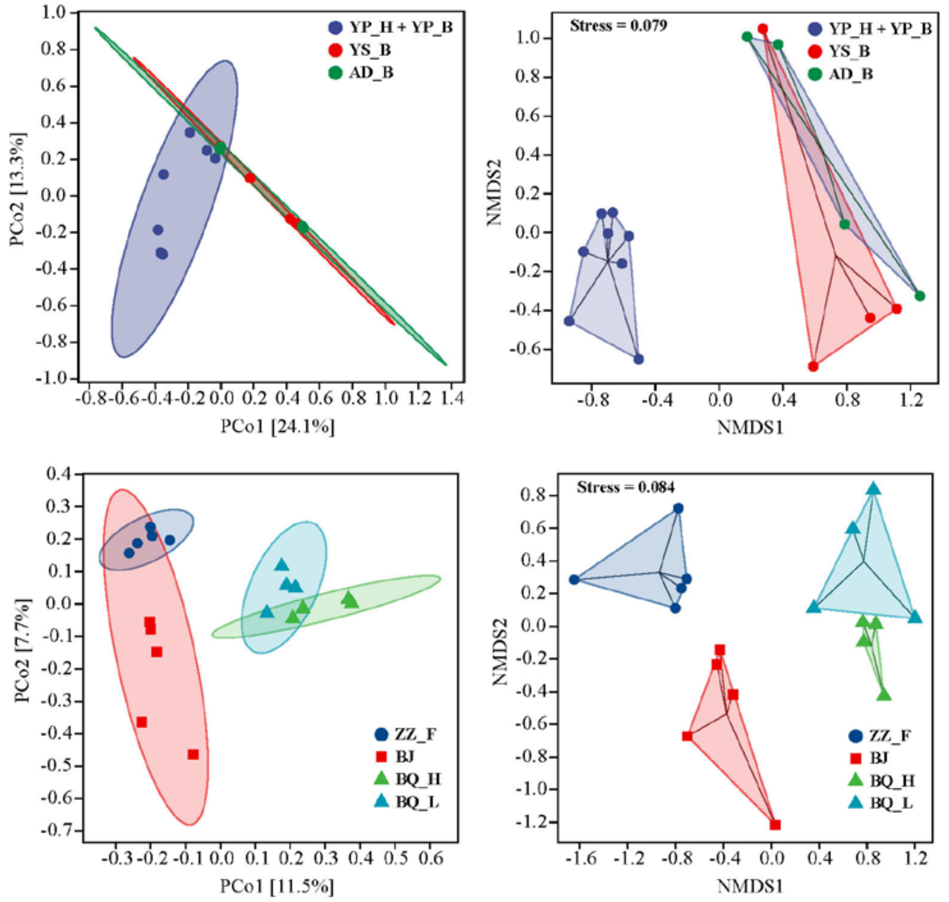

Figure S2 Comparisons of beta diversity among populations sampled in different sites and/or years, based on PCoA (left) and NMDS (right) using Bray–Curtis distance.

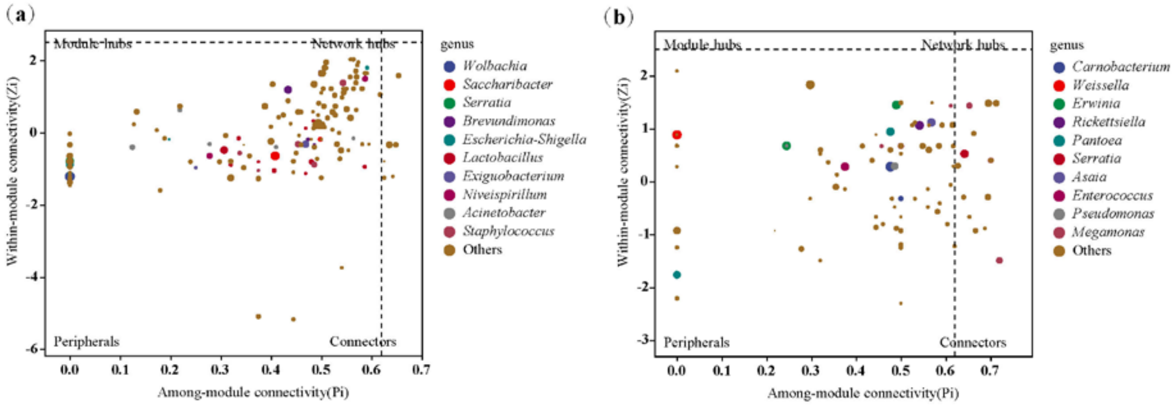

Figure S3 ZP-plots indicating the potentially keystone microbial taxon for representative *Parnassius* species of the subgenus *Parnassius* (host-plant: Crassulaceae + Saxifragaceae, left) and others (host-plant: Papaveraceae, right), according to the distribution of ASVs based on their module-based topological roles. Each dot represents an ASV in the dataset of different genera. The topological role of each ASV was determined according to the scatter plot of within-module connectivity (Zi) and among-module connectivity (Pi).

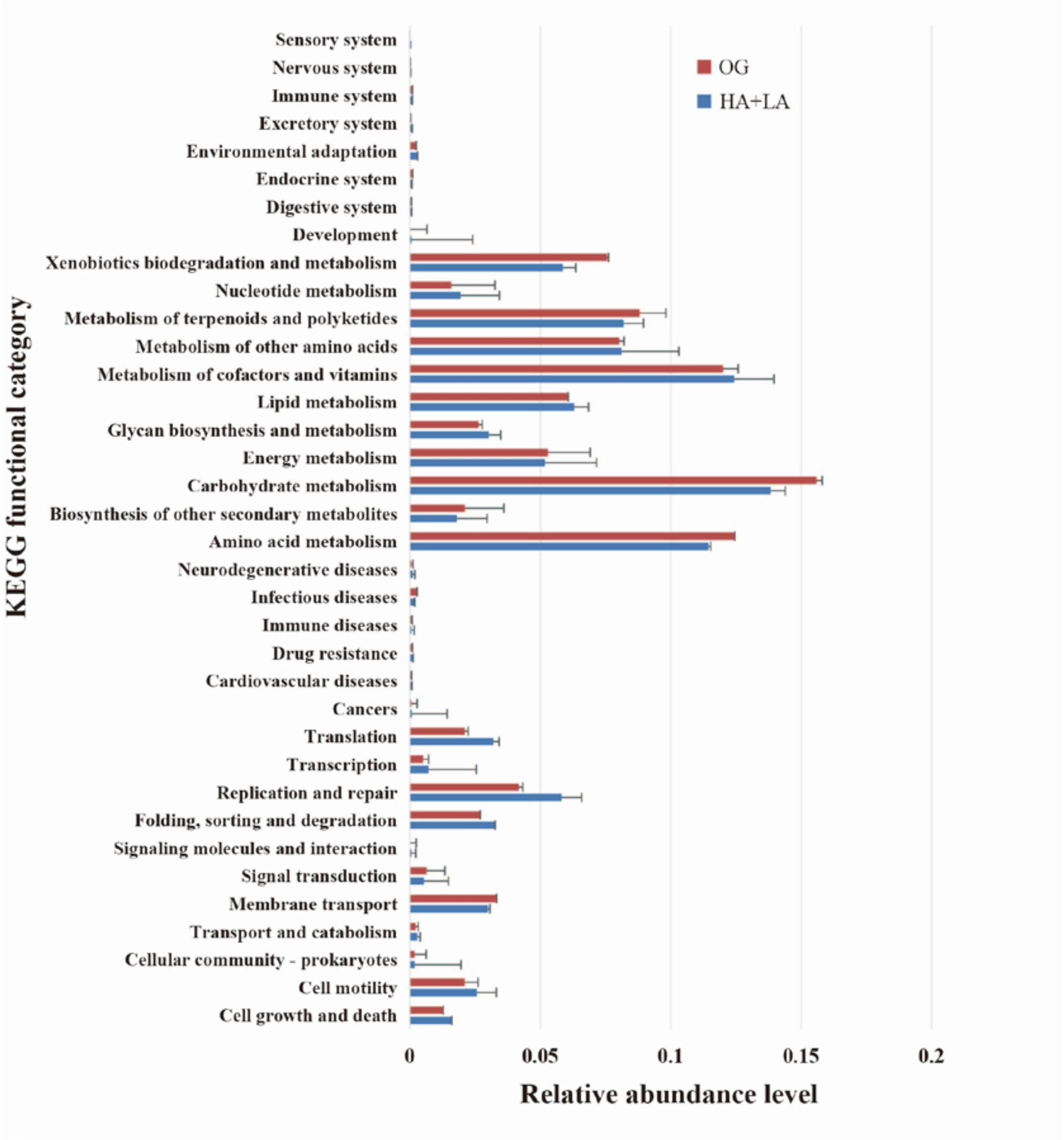

Figure S4 Result of KEGG functional prediction for the gut microbiota based on PICRUSt2, shown in relative abundance level.

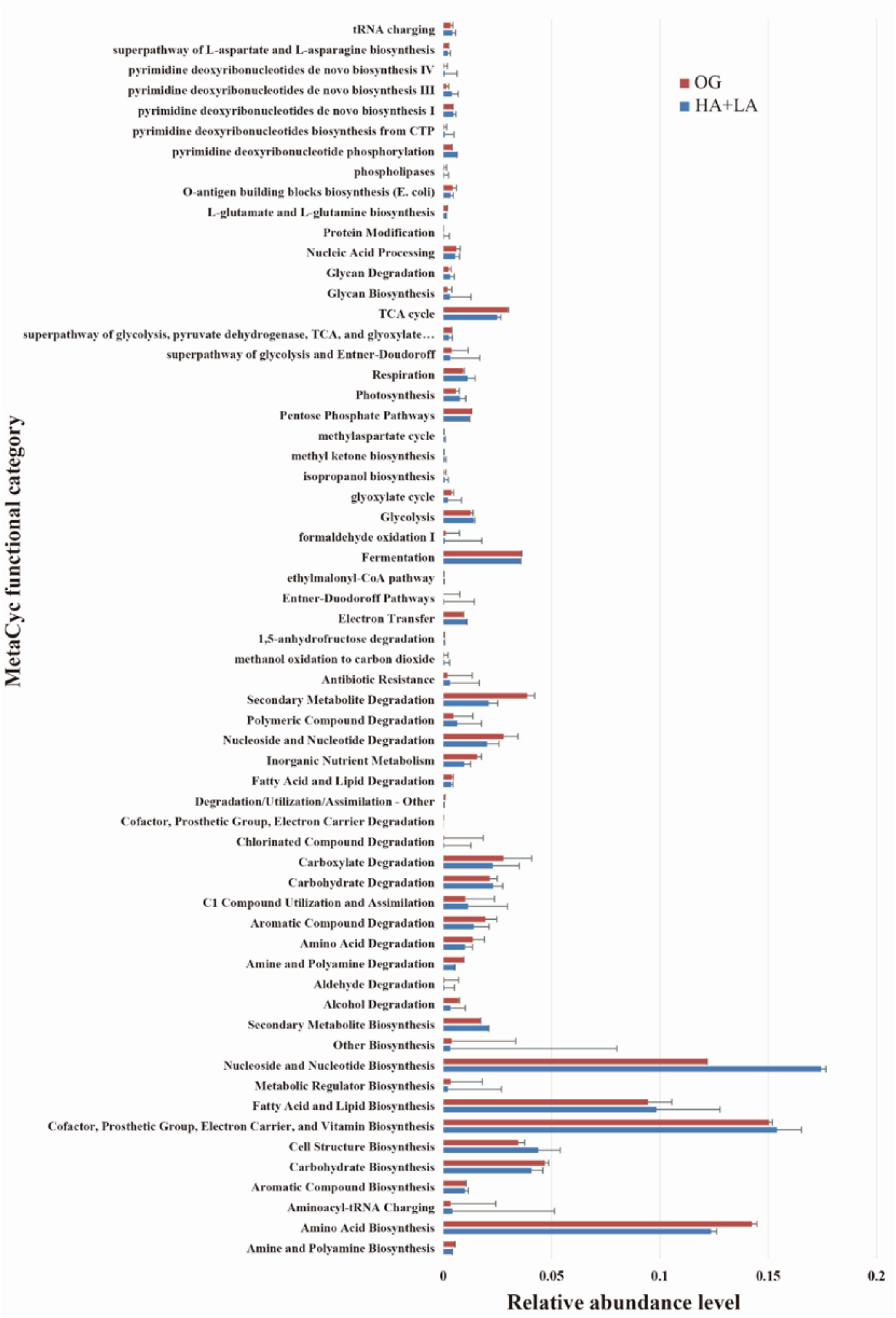

Figure S5 Result of functional prediction for the gut microbiota based on the MetaCyc database, shown in relative abundance level.
